# Characterisation of the Interaction among Oil-In-Water Nanocapsules and Mucin

**DOI:** 10.3390/biomimetics5030036

**Published:** 2020-07-28

**Authors:** Mar Collado-González, Gurmeet Kaur, Yadira González-Espinosa, Rebecca Brooks, Francisco M. Goycoolea

**Affiliations:** School of Food Science & Nutrition, University of Leeds, Leeds LS2 9JT, UK; G.Kaur@leeds.ac.uk (G.K.); Y.GonzalezEspinosa@leeds.ac.uk (Y.G.-E.); fs16rahb@leeds.ac.uk (R.B.); F.M.Goycoolea@leeds.ac.uk (F.M.G.)

**Keywords:** mucin, oil-in-water nanoparticles, chitosan, stability, ζ-potential, quorum sensing

## Abstract

Mucins are glycoproteins present in all mucosal surfaces and in secretions such as saliva. Mucins are involved in the mucoadhesion of nanodevices carrying bioactive molecules to their target sites in vivo. Oil-in-water nanocapsules (NCs) have been synthesised for carrying *N,N′*-(di-m-methylphenyl)urea (DMTU), a quorum-sensing inhibitor, to the oral cavity. DMTU-loaded NCs constitute an alternative for the treatment of plaque (bacterial biofilm). In this work, the stability of the NCs after their interaction with mucin is analysed. Mucin type III from Sigma-Aldrich has been used as the mucin model. Mucin and NCs were characterised by the multi-detection asymmetrical flow field-flow fractionation technique (AF4). Dynamic light scattering (DLS) and ζ-potential analyses were carried out to characterise the interaction between mucin and NCs. According to the results, loading DMTU changes the conformation of the NC. It was also found that the synergistic interaction between mucin and NCs was favoured within a specific range of the mucin:NC ratio within the first 24 h. Studies on the release of DMTU in vitro and the microbial activity of such NCs are ongoing in our lab.

## 1. Introduction

Dental caries is caused by the destruction of enamel, dentin, and dental pulp [1] due to state of dysbiosis among various bacterial and yeast species present in the buccal cavity. *Streptococcus mutans* has been reported as one of the primary cariogenic pathogens leading to the biofilm formation (dental plaque) further progressing into carious lesions (dental caries). Chronic dental infections lead to periodontitis, resulting in systemic inflammation that may manifest into chronic infective endocarditis [2] and rheumatoid arthritis [3], among other diseases [4].

In the oral cavity, bacteria and yeast are found in multispecies colonies that are called biofilms [1]. Such biofilms confer protection to the microbial organisms from various adverse environmental conditions such as antibiotics, thus resulting in the development of multi-drug-resistant bacterial species. Quorum sensing (QS) is among one of the various systems that is involved in the formation of biofilms by *S. mutans*. QS can be defined as cell-to-cell communication in bacteria through various small chemical moieties (in Gram-negative bacteria) and small peptides (in Gram-positive bacteria), resulting in expression of virulence (toxin production, competence development, biofilm formation, etc.) [2]. Inhibition of virulence through disruption of QS has been recognised among the potential ways to combat drug resistance without killing the pathogen and avoiding population stress in bacteria. This gives essential time to the host immune system to fight against the infection and serve as a means of additional therapy in combination with antibiotics. Additionally, disruption of the dental plaque (biofilms) can be potentially achieved by using antibacterial molecules loaded in delivery nanosystems [1], applying strategies to promote the inhibition of biofilm formation by exploiting QS [5] or by the application of low-molecular-weight chitosan (CS), for example [1].

Due to the microbial resistance to antibiotics, the development of alternative therapies, such as inhibition of QS, is of utmost interest [6]. We have recently uncovered the potent QS inhibition activity of a newly synthetic molecule *N,N′*-(di-m-methylphenyl)urea, also known as 1,3-dim-tolylurea (DMTU) [2]. DMTU interacts with glutamine 11, one of the amino acids in the active site of the peptidase (PEP) domain of ComA, which acts as an ABC transporter involved in the QS system of *S. mutans*. Thus, DMTU blocks the expression of virulence factors controlled by the QS system, such as the biofilm formation [2]. Besides, according to in vitro assays, DMTU down-regulates some genes involved in the expression of various virulence factors regulated through QS in *S. mutans* [7].

Oil-in-water nanocapsules (o/w NCs) prepared by solvent displacement enhance the bioavailability of lipophilic drugs [8]. Besides, o/w NCs have been evaluated for treating oral infections when carrying different bioactive molecules because they can adhere to tooth and mucosa, thus releasing their payload [6,9,10]. Vidal-Romero and colleagues synthesised o/w NCs that were applied as pills that melt in the mouth, for treating patients diagnosed with periodontitis. According to their results, their o/w NCs loaded with chlorhexidine significantly reduced the oral plaque in comparison to chlorhexidine mouthwash [11].

It has been reported that o/w NCs interact with the mucosa and biofilms and that such interaction is enhanced by polycationic o/w NCs [12,13] as they could interact electrostatically with bacterial membranes that are negatively charged. Thus, different polycationic polymers have been used for coating nanostructures such as Eudragit^®^ [13] or chitosan [14]. Chitosan (CS) is a family of polycationic biopolymers obtained from the deacetylation of chitin. CS can interact with mucin [15,16], the main glycoproteins in saliva and mucosal surfaces [15,17]. The structural characteristics of CS (degree of acetylation, DA, and molecular weight, MW) directly determine the ability to interact with mucin and reduce its viscosity [16]. NCs coated with lecithin and chitosan have been thoroughly studied as drug delivery platforms of biologics and lipophilic molecules [18,19] Recombinant hepatitis B surface antigen (HB) has been associated ionically at the surface of these systems and found to modulate the systemic immune response upon intra-nasal vaccination [20]. Yet, another asset of these systems is their stability in biological media determined by the careful selection of the DA and degree of polymerisation (DP) of chitosan [21]. We have shown that o/w NCs are an effective platform to load and deliver lipophilic drugs such as capsaicin [22] and triclabendazole [23]. In addition, it has been reported that CS has antibacterial activity on bacterial biofilms [1]. Thus, DMTU-loaded o/w NCs coated with chitosan might offer a synergistic effect against biofilms. Müller and colleagues reported the success of mouthwash formulations that showed antimicrobial activity while not impairing the cell viability of different oral cell lines in vitro [24]. Understanding how chitosan-based nanoparticle systems interact with mucosa is essential to their optimisation and fully realises their potential in drug delivery in various contexts.

Based on all the above, this study evaluated a potential new treatment against biofilms that consists of CS-coated DMTU-loaded o/w NCs and mucin. The reason behind this study is supported by three facts: First, in the oral cavity, o/w NCs interact first with saliva that covers all mucosal surfaces and teeth; second, mucins are the most abundant glycoproteins in the saliva [25]; and third, the nanosize and great stability offered by o/w NCs will guarantee their success for delivery. Thus, studying the evolution of size and stability is of utmost importance in the development of nanometric delivery systems. In this work, we evaluate the size of o/w NCs after the interaction with mucin molecules and the stability of the nanostructures developed. For this purpose, mucin type III from Sigma Aldrich has been used as a model of salivary mucin due to its structural and conformational similarities [25,26]. Despite there being two different types of porcine stomach mucin available at Sigma-Aldrich, mucin type III has been used in this work as it is obtained from mucin type II after purification.

## 2. Materials and Methods

### 2.1. Materials

Lecithin (Epikuron 145V) was kindly provided by Cargill. Miglyol 812 was purchased from Sasol GmbH (Hamburg, Germany). Hydrochloric acid, absolute ethanol, 1,3-di-m-tolylurea (DMTU, R395609-1EA), and mucins were purchased from Sigma Aldrich-Merck (Darmstadt, Germany). Chitosan was a sample of biomedical grade from Heppe Medical Chitosan GmbH (Halle, Germany) with the following specifications: Sample code 80/20 (batch Nr 212-201015-04), degree of N-acetylation ~20% (as determined ^1^H NMR), molecular weight of ~97.7 kDa, and polydispersity (PD) (PD = Mw/Mn) ~ 1.4 (determined by Size Exclusion Chromatography-Multiangle Light-Detector Refractive Index, SEC-MALS-DRI).

### 2.2. Methods

#### 2.2.1. Preparation of Oil-In-Water Nanocapsules

The synthesis of o/w NCs was performed following the methodology from Qin and colleagues with some modifications [27]. In brief, for preparing the organic phase, 400 µL of a solution of lecithin 100 g/L in ethanol, 530 µL of ethanol containing DMTU or absolute ethanol in the case of the preparation of blank o/w NCs, and 125 µL of miglyol 812 were all added in a beaker. After that, 9.5 mL of ethanol were added and mixed. This organic phase was poured on the top of 20 mL of chitosan solution. Then, the volume of the suspension was reduced to 10 mL by a rotary evaporator at 40 °C and slow-speed gyration to allow the solvents to evaporate without boiling the suspension. Once at room temperature, suspensions were kept at 4 °C until further use.

#### 2.2.2. Preparation of Mucin Solutions

The hydrophilic mucins were isolated as explained elsewhere [16]. Briefly, commercially available mucins were dissolved at 5 mg/mL in MilliQ water by stirring overnight. Mucin solution was centrifuged at 25.000× *g* and 10 °C for 50 min. Supernatants were collected and 0.02% *w/v* sodium azide was added. The main goal of this process is to purify the free mucin particles in the mucin suspension. Then, mucin solution was aliquoted and freeze-dried. By subtracting the weight of the samples before and after freeze-drying, the amount of mucin was accurately determined. Then, lyophilised mucin was stored until further use. In that moment, the mucin sample was diluted up to 5 mg/mL and stirred at least 3 h to achieve full dissolution of the glycoprotein. Once dissolved, the mucin solution was serial-diluted by a factor of 2 until a concentration equal to 0.15625 mg/mL was reached. The range of mucin concentrations used included the mucin concentration in saliva, that is, 36% *w*/*w* [28].

#### 2.2.3. Interactions Nanocapsules—Mucin

For the analyses of the interaction between o/w NCs and mucin, 0.1 mL of NCs was added to 0.9 mL of a mucin solution at 5, 2.5. 1.25, 0.625, 0.313, or 0.156 mg/mL. To simplify the notation, the final mass of the o/w NCs and the mucin on the aliquots was calculated, and the samples are noted as a mucin:o/w NC ratio in *w*/*w*. Thus, the mucin:o/w NC ratio in *w*/*w* corresponding to the mucin solutions indicated above are: 2.68, 1.34, 0.67, 0.34, 0.17, and 0.08, respectively.

Three different aliquots were prepared: One of them was analysed after adding o/w NCs in mucin solution and corresponds to 0 h, and the other two samples were kept at 37 °C to be analysed after 1 and 24 h. The hydrodynamic size of the particles in all the samples was analysed by dynamic light scattering (DLS), while the ζ-potential was measured only in samples analysed at 0 and 24 h.

#### 2.2.4. Dynamic Light Scattering (DLS) Analysis

Hydrodynamic size and ζ-potential were measured by using a Zetasizer Ultra (ZSU5700, Malvern instruments, Worcestershire, UK). Hydrodynamic size was measured by analysing the back-scattered light (173°) from the sample. The attenuator and the position of the photoreceptor was fixed by software in an automatic mode. The working temperature was fixed to 25 °C. Once the sample was introduced in the chamber, the time for the temperature stabilisation was 120 s. Then, the size was measured in triplicate, followed by the analysis of the ζ-potential, with no extra time for equilibrating the temperature of the sample. The ζ-potential was measured in triplicate. All the curves included in this paper correspond to the average of the measurements. The analysis of the particle size was performed by Multiple Narrow mode for mucin suspensions and General Purpose mode for NCs. The size distribution by intensity was used for the analysis of the samples. The polydispersity index was obtained by cumulant analysis and it is included for comparative purposes only. As some of the suspensions showed a higher polydispersity index than 0.5, the size included in this work results from the distribution analysis from the distribution by intensity.

#### 2.2.5. Asymmetrical Flow Field-Flow Fractionation (AF4)—Multidetector Analysis

Physico-chemical characterisation of mucins was performed by asymmetrical flow field-flow fractionation, AF4, using an AF200 Multiflow system (Postnova Analytics UK Ltd. Malvern, UK) coupled [29] with an autosampler (PN5300), isocratic pump (PN1130), solvent organiser (PN7140), solvent degasser (PN7520), UV disinfection (PN7205), RI detector (PN3150), MALS detector (PN3621), and UV detector (SPD-20A). For facilitating ease of reading, the equipment will be denoted as AF4. The measurement was performed using a regenerated cellulose membrane (Z-AF4-MEM-612-10KD, Postnova Analytics UK Ltd. Malvern (Worcestershire, UK)) with a 10 kDa cutoff. The spacer was 350 µm and the temperature was set to 30 °C by a thermostat (PN4020). Data collection and analysis were performed using NovaFFF software version 2.0.9.9 Postnova Analytics UK Ltd. (Worcestershire, UK).

For the analysis of mucin, 13 µL of mucin type III at 5 mg/mL concentration of NaCl 0.01 M were injected using NaCl 0.01 M as a carrier liquid filtered through a 0.1 µm membrane. The system was set-up at a constant detector flow rate of 0.5 mL/min, the flow rate of the focus was 2.5 mL/min, and the focusing time was 3 min. The elution was set as follows: A constant elution at 2.5 mL/min of cross-flow for 0.2 min. Then, the cross-flow was reduced to 0.2 mL/min in a power decay mode with an exponent of 0.25 in 20 min and then reduced to 0.12 mL/min in 5 min with an exponent of 0.8. Finally, the cross-flow was reduced up to 0.09 mL/min in 5 min with an exponent of 0.8. The signal of a blank consisting of 13 µL of NaCl 0.01 M injection was recorded using the same method and, for analysis, subtracted from the recorded signals obtained from mucins.

For the AF4 analysis of mucin, dn/dc was 0.144 according to Maleki and colleagues [30]. MALS angles included in the analysis corresponded only to those between 44° and 140°, as poor signals were detected from lower and higher angles. Data recorded were fitted to a random coil model.

The radius of gyration (R_g_) can be obtained from MALS analysis while the hydrodynamic radius (R_h_) can be obtained from DLS measurements. The conformation of the mucin molecules, therefore, can be determined from the shape factor (*ρ*) that is calculated as indicated in Equation (1).

(1) $$\rho =\frac{R_{g}}{R_{h}}$$

Soft spheres are defined by *ρ* = 0.5, hard spheres by *ρ* = 0.77, coated spheres by *ρ* close to 1, oblate spheroids by *ρ* higher than 1, and prolate spheroids by *ρ* higher than 2 [31,32].

## 3. Results

### 3.1. Mucin Characterisation

**Figure 1:**
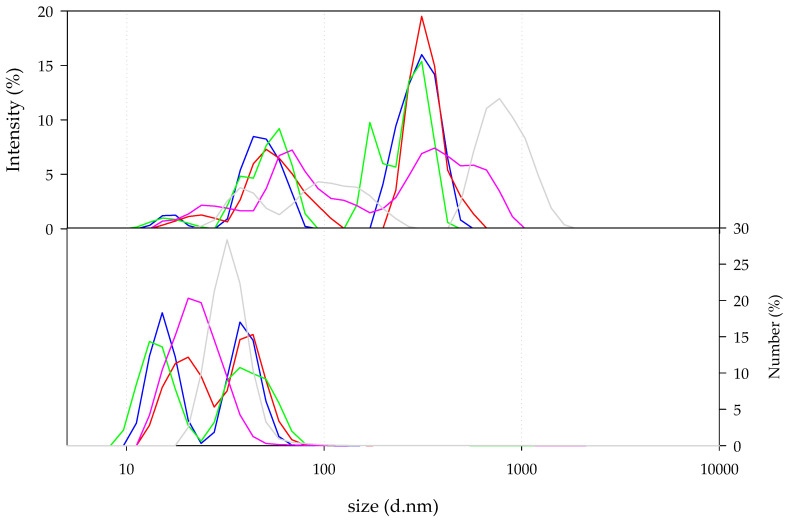
Hydrodynamic diameter of mucin type III from Sigma-Aldrich at 1.25 (blue), 0.625 (red), 0.313 (green), 0.156 (pink), and 0.078 mg/mL (grey). Size distribution by intensity (**top**) and by number (**bottom**).

**Figure 2:**
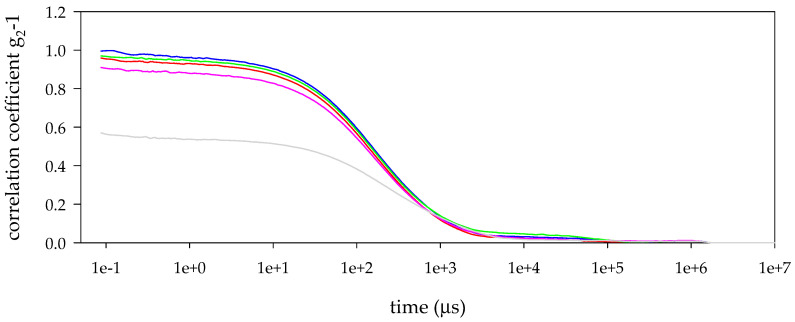
Correlation function of mucin type III from Sigma-Aldrich at 1.25 (blue), 0.625 (red), 0.313 (green), 0.156 (pink), and 0.078 mg/mL (grey).

Dynamic light scattering (DLS) analyses of mucin type III from Sigma-Aldrich revealed the presence of populations with different hydrodynamic diameters. According to the size distribution by intensity (Figure 1, top), the smallest population appears in the range between 10 and 30 nm. The second population appears in the range from 30 to 100 nm, approximately. In addition, the third population of mucin particles appears in the range from 100 to 1000 nm. It is important to note that decreasing the concentration of mucin solutions, below 0.313 mg/mL, resulted in the movement of populations toward higher hydrodynamic diameter ranges according to profiles of size distribution by intensity. The 0.078 mg/mL mucin solution can be described by the presence of three peaks. The main population centred around 846 nm of hydrodynamic diameter, the second one centred around 119 nm, and the last one centred around 39 nm of hydrodynamic diameter. The reduction in the mucin concentration resulted in a poor signal recorded due to the low number of molecules in the solution that resulted in a decrease in the correlation coefficient (Figure 2). According to the physics principles of the DLS technique and as it can be seen in the size distribution by number (Figure 1, bottom), the population of particles higher than 100 nm is negligible in any of the mucin solutions.

**Figure 3:**
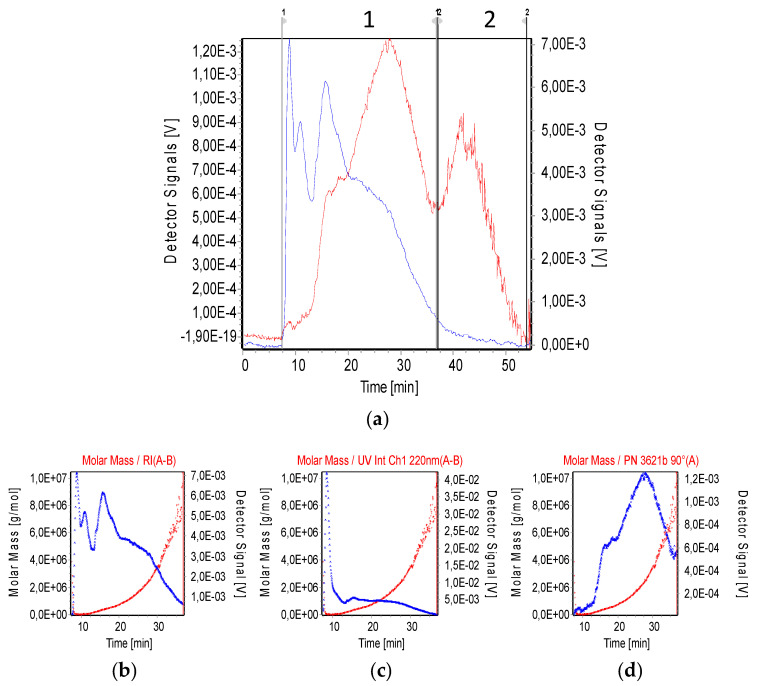
Measurement of mucin by asymmetrical flow field-flow fractionation (AF4); refractive index (blue) and MALS at 90° (red) signals recorded by AF4 of mucin type III from Sigma Aldrich (**a**). Analyses of region 1 of the recording: Mucin: RI (**b**), UV absorbance at 220 nm (**c**), and light scattering at 90° (**d**). In all the analyses, molar mass is included.

Mucin was analysed by AF4. The elution of the mucin molecules is determined by the MALS and UV absorbance at 220 nm signals while the RI signal gives an indication of the concentration of analyte present in the sample (Figure 3). The MALS signal associated with the size of eluting material shows two prominent peaks. The first one where the intensity of the RI signal is predominant indicates a higher concentration of eluting material. However, the intensity of the RI signal over the second peak of MALS is significantly low, indicating that there is a fraction of a bigger-size material but of negligible concentration. This can correspond to the presence of aggregates present in solution. Analysis for the calculation of MW was, therefore, determined over the region where the material was assumed to be soluble (region 1, as delimited by integration limits in Figure 3).

The analysis of regions 1 and 2 of the AF4 recording is depicted in Table 1. The low mucin recovery after the measurement could be due to possible interactions of the mucin with the membrane, even though the AF4 membrane was negatively charged. The interaction hypothesis is reinforced by the noisy signal from the MALS at low angles (not shown). The *ρ* was calculated from the gyration radii (Rg) obtained from AF4 and the R_h_ obtained for each population of mucin at 5.0 mg/mL obtained by DLS (Table S1). The *ρ* of mucin molecules in region 1 of AF4 analysis, which corresponds to the population centred around 40 nm in hydrodynamic radii in DLS recordings, was 1.26, which corresponds to oblate spheroids particles. Meanwhile, the *ρ* for those particles found in region 2 in the AF4 analysis and centred around 270.2 nm of hydrodynamic radii in DLS measurements was 0.93, which corresponds to a coated sphere. Such a change in the conformation of the mucin in the solution reinforces the hypothesis of the mucin aggregation.

### 3.2. Characterisation of Oil-In-Water Nanocapsules

We have formulated o/w NCs for the delivery of DMTU, which shows antibiofilm properties, and it is of potential use for the prevention of dental caries.

**Figure 4:**
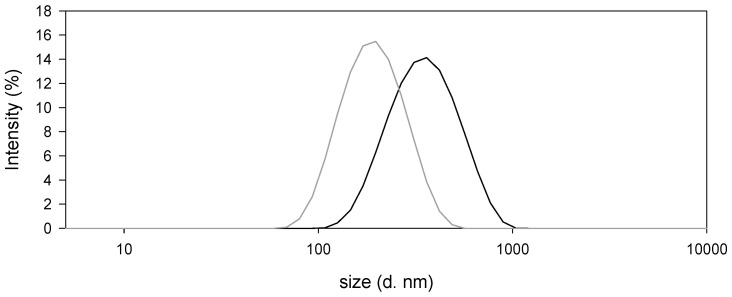
Size distribution by intensity of blank o/w nanocapsules (NCs) (grey) and 1,3-di-m-tolylurea (DMTU)-loaded o/w NCs (black).

The inclusion of the DMTU in the formulation resulted in the increment in the hydrodynamic size, as shown in distributions by intensity of the o/w NCs obtained (Figure 4). For comparison, blank o/w NCs (B o/w NCs) showed a z-average hydrodynamic diameter equal to (176 ± 2) nm with a polydispersity index (PDI) of 0.13, while DMTU-loaded o/w NCs (P o/w NCs) showed a z-averaged hydrodynamic diameter of (287.3 ± 6.4) nm with a PDI of 0.25 (Figure 4). Regarding the ζ-potential, both formulations were positively charged, indicating the deposition of chitosan in their outer part. The ζ-potential values were +40 and +54 mV for B o/w NCs and P o/w NCs, respectively.

### 3.3. Interaction among NCs and Mucin

Due to the negative charge of the mucin molecules in solution, (−16 ± 1) mV, an interaction is expected between these and the chitosan that is coating the o/w NCs, via the electrostatic interactions, in addition to the likely existence of other interactions between both. In addition, we have analysed the effect of these interactions on the stability of the o/w NCs. We define stability as the maintenance of the nanometric size of the o/w NCs.

#### 3.3.1. Interaction among Blank Nanocapsules and Mucin

**Figure 5:**
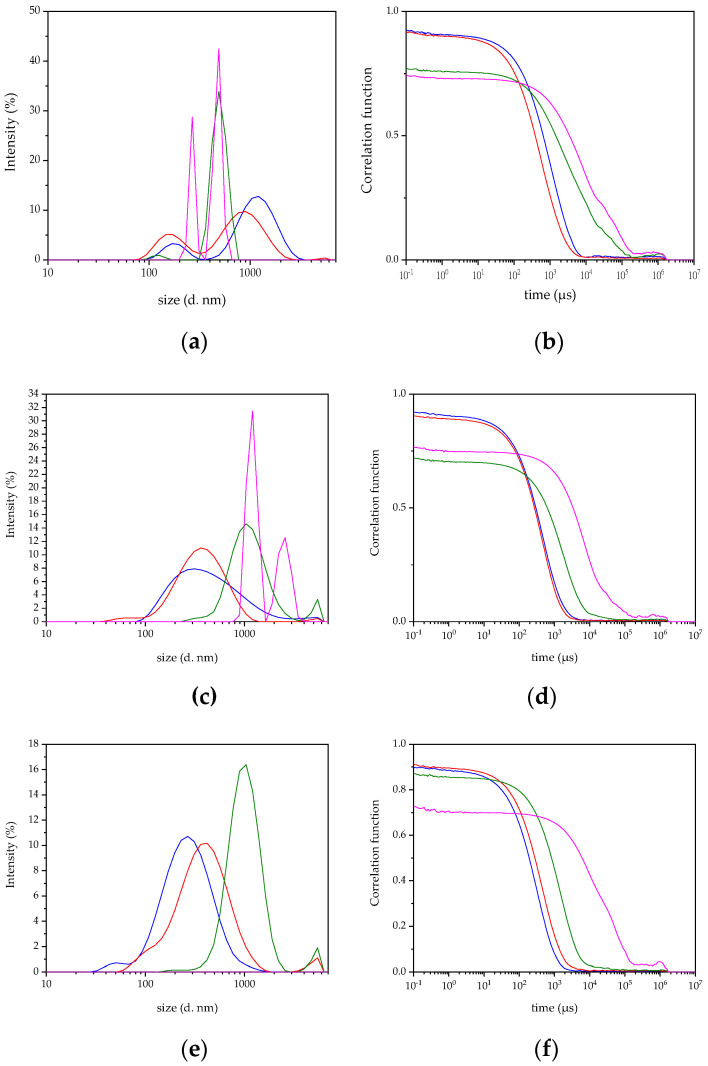
Size analyses of the nanostructures obtained after adding blank o/w NCs to mucin solutions at mucin:B o/w NC ratios of 0.08 (*w*/*w*) (pink), 0.17 (*w*/*w*) (green), 0.34 (*w*/*w*) (red), and 0.67 (*w*/*w*) (blue): (**a**) Comparison of hydrodynamic diameter and (**b**) correlogram functions of the suspensions at time 0 h; (**c**) comparison of hydrodynamic diameter and (**d**) correlogram functions of the suspensions after 1 h of incubation at 37 °C; (**e**) comparison of hydrodynamic diameter and (**f**) correlogram functions of the suspensions after 24 h of incubation at 37 °C.

**Figure 6:**
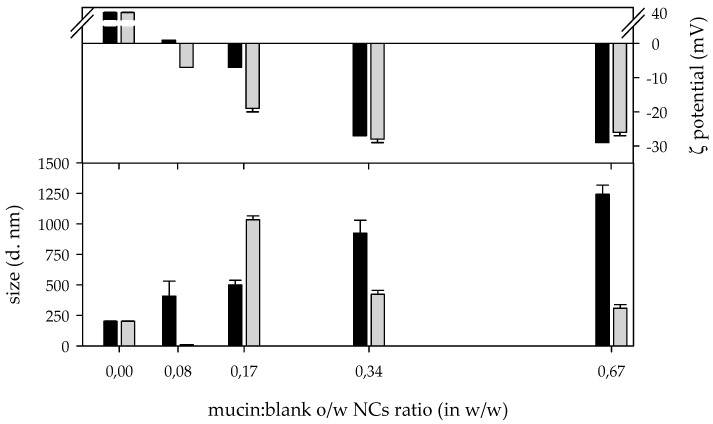
ζ-potential and size (Z-average) of nanocomposites (*n* = 3) obtained after the interaction among mucin and blank nanocapsules at different ratios at 0 (black) and 24 h (grey) after the preparation.

The interaction among mucin and B o/w NCs resulted in nanocomposites (NCOMs) of different hydrodynamic diameters depending on the mucin:B o/w NC ratios (in *w*/*w*). The interaction among mucin and B o/w NCs at a mucin:B o/w NC ratio equal to or below 0.17 *w*/*w* resulted in a NCOM larger than the detection limit of the DLS technique as it could be interpreted from the sharp peaks in Figure 5a and the correlation functions in Figure 5b. In those functions, it is noteworthy that the decrease in the intercept value, which can be interpreted as the decrease in the number of particles in the suspension, resulted from the aggregation of the NCOMs. Moreover, the low ζ-potential in absolute values of such NCOMs indicates that the electrostatic repulsive forces that keeps the NCOMs away from each other must be weak (Figure 6 and Table S2). At a higher mucin:B o/w NC ratio, two populations of NCOMs were obtained, one of them centred on hundreds of nm, which is the most abundant, and the second population centred on thousands of nm (Figure 5a).

After 1 h of incubation at 37 °C, those NCOMs prepared at a mucin:B o/w NC ratio of 0.08 (*w*/*w*) still showed micrometric size (Figure 5c), although the hydrodynamic size of the NCOMs in this solution was out of the detection limit of the DLS technique (Figure 5d). All the NCOM suspensions prepared with a higher amount of mucin showed one broad peak centred at different hydrodynamic diameters. Thus, NCOMs prepared at a mucin:B o/w NC ratio of 0.17 (*w*/*w*) were centred on thousands of nm, while those prepared at mucin:B o/w NC ratios of 0.34 (*w*/*w*) and 0.67 (*w*/*w*) showed populations centred on hundreds of nm (Figure 5c). After 24 h, those NCOMs prepared at a mucin:B o/w NC ratio of 0.08 (*w*/*w*) were completely out of the detection limit, as it could be inferred from the correlogram function (Figure 5f) and the absence of peaks in the intensity distribution (Figure 5e), whilst the rest of the NCOM suspensions showed their main peaks in the same region of hydrodynamic diameter and did not show strong changes in the size of the main population with respect to the measurement at 1 h. However, it is important to point out that all the curves showed a second peak or a shoulder in the region of lower hydrodynamic size.

#### 3.3.2. Interaction among DMTU-loaded o/w NCs and mucin

**Figure 7:**
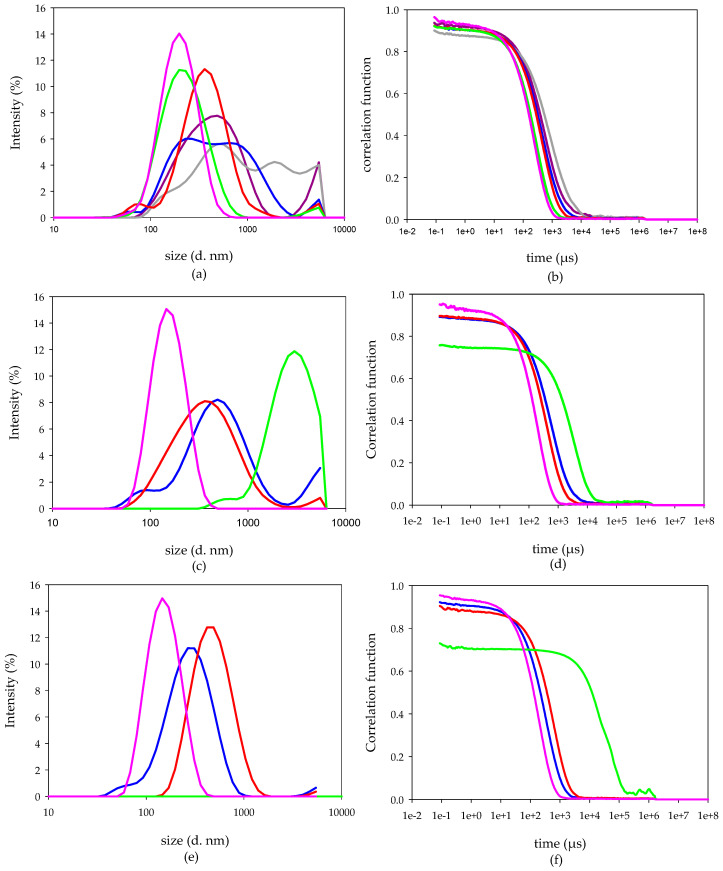
Hydrodynamic diameter distribution by intensity of nanocomposites obtained after adding DMTU-loaded o/w NCs to mucin solutions at mucin:P o/w NC ratios of 0.08 (*w*/*w*) (pink), 0.17 (*w*/*w*) (green), 0.34 (*w*/*w*) (red), 0.67 (*w*/*w*) (blue), 1.34 (*w*/*w*) (grey), and 2.68 (*w*/*w*) (purple): (**a**) Comparison of hydrodynamic diameter and (**b**) correlogram functions of the suspensions at time 0 h; (**c**) comparison of hydrodynamic diameter and (**d**) correlogram functions of the suspensions after 1 h of incubation at 37 °C; (**e**) comparison of hydrodynamic diameter and (**f**) correlogram functions of the suspensions after 24 h of incubation at 37 °C.

Similar to the results obtained previously, the interaction among mucin and P o/w NC yield NCOM depended on the mucin:P o/w NC ratios (in *w*/*w*). At mucin:P o/w NC ratios up to 0.17 (*w*/*w*), the hydrodynamic diameter of the NCOMs obtained was smaller (centred on 200 nm) than that of the P o/w NCs. The hydrodynamic diameter increased when increasing the amount of mucin in the formulation. Thus, those NCOMs obtained at a mucin:P o/w NC ratio of 0.34 (*w*/*w*) showed two populations, one of them centred around 80 nm and the second one centred around 360 nm (Figure 7a). Then, including higher amounts of mucin in the formulations resulted in an increase in both the size and the polydispersity of the NCOMs obtained. At a mucin:P o/w NC ratio of 0.67 (*w*/*w*), two populations of particles were recorded, one of them centred in the hundreds of nm and the second one centred in the thousands of nm. At a mucin:P o/w NC ratio higher than 0.67 (*w*/*w*), just one broad population spans from hundreds to the out-of-detection limit of the technique (Figure 7a). According to the correlation function of these systems, the measurements carried out had good quality (Figure 7b).

**Figure 8:**
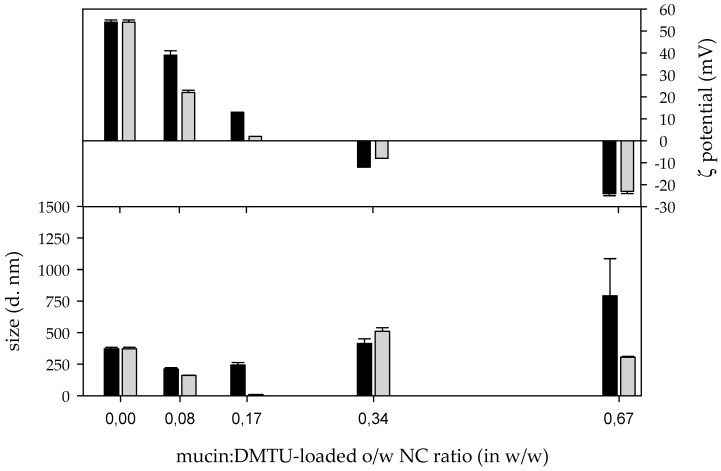
ζ-potential and size (Z-average) of nanocomposites obtained after the interaction among mucin and DMTU-loaded nanocapsules at different ratios at time of synthesis (black) and 24 h (grey) after the preparation event.

Regarding their ζ-potential measurements, NCOMs prepared at a mucin:P o/w NC ratio of 0.08 (*w*/*w*) had a ζ-potential lower than that of P o/w NCs (Figure 8 and Table S3), indicating that those NCOMs had enough mucin to modify the net charge of the P o/w NCs but not enough to revert their charge. Increasing the amount of mucin in the system up to a mucin:P o/w NC ratio of 0.34 (*w*/*w*) resulted in unstable NCOMs according to their ζ-potentials. Repulsive electrostatic forces in those nanoparticles with a ζ-potential lower than 25 mV in absolute value are weak and cannot avoid the aggregation of the nanoparticles [33,34]. Interestingly, while the NCOMs obtained in the mucin:P o/w NC system with a ratio of 0.17 (*w*/*w*) were positively charged, those prepared at a ratio of 0.34 (*w*/*w*) were negatively charged, indicating that the charge balance among CS and mucin had been overcome in the latter. Then, increasing the amount of mucin in the system, the ζ-potential values of the P o/w NCs coated with mucin reached a plateau stage at a mucin:P o/w NC ratio equal to or above 0.67 (*w*/*w*) (Figure 8, Table S3, and Figure S1). The NCOMs prepared at mucin:P o/w NC ratios of 1.34 (*w*/*w*) and 2.68 (*w*/*w*) were removed from further analyses due to the impossibility of checking the evolution of these systems. The size of all the rest of the NCOM systems was checked after being incubated at 37 °C. NCOMs prepared at a mucin:P o/w NC ratio of 0.08 (*w*/*w*) showed no change in the hydrodynamic size even after 24 h of being synthesized (Figure 7c,e). The constant hydrodynamic size of these NCOMs could be related to the fact that although their ζ-potential decreased once the mucin was added, it was within the stability region (Figure 8 and Table S3). The hydrodynamic diameter of the NCOMs prepared at a mucin:P o/w NC ratio of 0.17 (*w*/*w*) showed a strong size increment after 1 h of incubation, reaching micrometric size, as can be seen in the size distribution by intensity and by the correlation function of this suspension (Figure 7c,d). The aggregation process of these NCOMs continued and, after 24 h of incubation at 37 °C, these NCOMs became larger than the limit of detection of the technique, as can be seen from the correlogram (Figure 7f) and from the absence of any peak in the hydrodynamic diameter distribution by intensity (Figure 7e). Regarding the rest of the systems, NCOMs prepared at a mucin:P o/w NC ratio of 0.34 (*w*/*w*) showed no change after 1 h of incubation. However, enlarged nanoparticles were found after 24 h of incubation at 37 °C. Such an increment in the NCOM size could be related to their low ζ-potential (Figure 8 and Table S3). NCOMs prepared at a mucin:P o/w NC ratio of 0.67 (*w*/*w*) showed a reduction in their hydrodynamic diameter along the experiment time (Figure 7c,e), while their ζ-potential did not change since the synthesis (Figure 8 and Table S3).

## 4. Discussion

In this work, we have synthesised CS-coated DMTU-loaded o/w NCs for their potential use against biofilm-forming bacteria in oral treatment. Such a nanometric system can interact with mucin, the most abundant glycoproteins in saliva and mucosal surfaces [13]. Mucin type III from Sigma-Aldrich has been used as, in addition to its similarity with other mucin types, it offers homogeneity between mucin samples and constitutes the easiest way to obtain purified mucin.

To characterise macromolecules, it is important to work with free macromolecules in solution. Thus, it is important to work at a concentration below the overlapping concentration (c*) for the macromolecular molecules at defined conditions. Different c* has been reported for mucin, from c* = 0.1 [23] to 5 mg/mL [27]. Our results agree with the latter as our DLS results showed no difference in the size of the molecules at concentrations below 5 mg/mL.

Mucin molecules showed a R_h_ around 40 nm according to our DLS analysis and Rg around 51 nm according to our AF4. These results fit well with the R_h_ values found in the literature that spans from 44 nm determined by DLS to 75 nm determined by AF4 in similar conditions [30,35]. The molecular weight (Mw) of the porcine gastric mucin, determined in this work to be (1.21 ± 0.02) × 10^6^ g/mol, is in agreement with previous reports on mucin [36,37] but disagrees with the molecular weight reported by Maleki and colleagues (2008), who reported a value one order of magnitude higher than that found in this work. This could be due to the degradation of the mucin particles during the purification process, as hypothesised by Maleki and colleagues [30]. Most of the particles in the solution are free oblate-shaped mucin molecules according to their *ρ* = 1.27, which is consistent with elongated structures. This result is in agreement with the findings in the literature, where free mucin molecules have been described as rod-like structures [38]. In our results, a negligible population of aggregated mucin molecules can be detected according to AF4 measurements and the decrease in the *ρ*.

In this work, commercially available mucin solutions have been prepared at concentrations between 0.15 and 5 mg/mL, as at this concentration, mucin is present as fully dissolved polymer (free mucin molecules). Moreover, similar concentration ranges have been previously reported in the literature on mucin interactions in binary systems [26,39].

Oil-in-water NCs, prepared by the solvent displacement method, showed a nanometric size as expected because of the use of Miglyol 812 as a lipid phase and lecithin as a surfactant [8], and the methodology used for their synthesis [12,27,40]. The electrostatic interaction among the polycationic CS and negative phosphatidic groups from the lecithin [8,41] results in the adsorption of CS on the surface of o/w NCs. According to previous studies in which equivalent nanocapsules were prepared, it was shown that more than 96.8% of CS was incorporated in the nanocapsules [21]. Therefore, the amount of free CS in the system and its interaction with mucin in the current work can be considered neglectable. CS also adsorbs on other types of surfactant used for the preparation of o/w NCs, such as Tween 80. It has been reported that such o/w NCs had a ζ potential lower (+17 ± 2) mV [40] than that of the o/w NCs prepared in the current research work in which the addition of DMTU resulted in a change in the ζ potential from (+40 ± 1) to (+54 ± 1) mV. In addition to the net charge change, the variation in the size of the o/w NCs after adding DMTU indicates a change in the conformation of o/w NCs.

The interaction among CS and mucin [42,43,44], as well as the interaction among CS-coated liposomes and mucin [45], has been reported, in addition to the favourable interaction of small-sized particles with mucosa [13]. Thus, the study of the interaction among the o/w NCs formulated and mucins continues to be of great interest to find out if such nanosystems are stable enough to resist their interaction with mucins. Therefore, they could be used as drug delivery systems in oral environments.

pH is an important factor in the interaction between polyelectrolytes such as chitosan and mucin. The final pH of the chitosan solution was 3.6 and the pH of the nanocapsules suspension was 2.5. On the other hand, the pH of the mucin solution was 7.0. Considering that the interaction between chitosan and mucin is favoured in the pH range from 2.4 to 6.5, and the pH of the interacting suspensions and the fact that only one population are found after their interaction, the most likely explanation is that the interaction between nanocapsules and mucin occurs via chitosan–mucin electrostatic attraction. The resulting structures obtained after the interaction among mucin and o/w NCs (NCOM) depended on the mucin: o/w NC ratio (in *w*/*w*), revealing that the interaction among the nanostructures occurred through electrostatic interactions. According to the results, the higher the content of mucin, the lower the ζ-potential of the NCOMs in both systems, B o/w NCs, and P o/w NCs. There are differences in the behaviour of both systems that differ in the absence or presence of DMTU. The balance among the charges among mucin and the B o/w NCs occurs at a mucin:B o/w NC ratio of 0.08 (*w*/*w*), approximately. Thus, the NCOMs prepared at such a ratio aggregated almost in the time of the interaction among the species. In addition, after 24 h, the particles in the suspension showed a negative charge. This could be explained as if mucin molecules were acting as glue among different o/w NCs. On the contrary, the interaction at mucin:B o/w NC ratios equal to or higher than 0.17 (*w*/*w*) resulted in small NCOMs at the time of synthesis as a result of the increase in the elasticity of the polyelectrolytes after their interaction [46] and the appearance of a compact layer of a polyelectrolyte adsorbed onto a layer of polyelectrolyte of opposite charge, for example, a layer of CS on a layer of mucin and vice versa [47]. The evolution of the different systems depended on the ζ potential of the system. At a mucin:B o/w NC ratio of 0.17 (*w*/*w*), a micrometric size after 24 h of incubation was shown due to the weakness of the electrostatic repulsive forces in the system. However, those systems prepared at mucin:B o/w NC ratios higher than 0.17 (*w*/*w*) were electrostatically stable, as is proven by their higher ζ potential. Therefore, those NCOMs were of nanometric size. Interestingly, the higher the amount of mucin, the smaller the size of the final NCOMs obtained.

Regarding the P o/w NCs, the balance of charges occurs in mucin:P o/w NC ratios among 0.17 (*w*/*w*) and 0.34 (*w*/*w*). Unlike the previous case, those NCOMs prepared at a mucin:P o/w NC ratio of 0.08 (*w*/*w*) showed no significant change in its size after 24 h. In such a system, the presence of mucin reduces both the ζ potentials and the size of the NCOMs after the interaction between the reactant species. At a mucin:P o/w NC ratio of 0.17 (*w*/*w*), aggregation occurred in a slow kinetic process. After 1 h, the system showed micrometric particles that underwent further aggregation up to be not detectable by the technique after 24 h of incubation. Again, the electrostatic interactions seemed to be responsible for the results obtained, as the ζ potential at the time of synthesis was +13 mV. Surprisingly, NCOMs showed that a low negative ζ potential, −12 mV, did not walk the same path despite the weakness of the electrostatic repulsive forces. Thus, those NCOMs prepared at a mucin:P o/w NC ratio of 0.34 (*w*/*w*) showed a nanometric population of particles after 24 h of incubation. In that time, the NCOMs had a ζ potential of +8 mV. The simplest explanation for that result is the presence of another type of stabilising force, such as steric forces, appearing among mucin molecules that are adsorbed on the surface of the NCOMs. Likely, such steric forces avoid the aggregation of the NCOMs in the system prepared at a mucin:B o/w NC ratio of 0.17 (*w*/*w*), which had −7 mV at the moment of the synthesis and −19 mV 24 h later, and the particles were still detectable by the technique. The increase in the mucin content up to a mucin:P o/w NC ratio of 0.67 (*w*/*w*) resulted similarly to the system prepared with B o/w NCs at the same ratio (in *w*/*w*). That is, as time passes, the size of the NCOMs decreases as a result of the increase in the flexibility of the polyelectrolytes after their interaction [46]. At higher mucin:P o/w NC (*w*/*w*) ratios, the final system resulted in higher structures at the time of synthesis. For example, the system prepared at a mucin:P o/w NC ratio of 1.34 (*w*/*w*) showed a broad population that spans from hundreds of nm up to micrometric-size particles, which fall out of the detection limit of this technique.

Few similar research works are available in the literature. Ünal et al. (2015) prepared o/w NCs in a similar way to those proposed in this work but using Tween 80 as a surfactant instead and Protasan UP CL 113 chitosan (DA = 10–25%, Mw = 50–150 kDa). The CS used in that work had characteristics similar to those of the polymer used in this study (DA = 20%, Mw = 97 kDa), so it is expected that the behaviour exhibited with mucin is similar. In the work from Ünal et al. (2015), the interactions among mucin and the o/w NC were evaluated at a mucin:o/w NC ratio of 0.03 (*w*/*w*) (value determined based on their used methodology) and analysed by turbidity. According to their results, there was a significant interaction between chitosan-coated nanoparticles and mucin; however, no comparisons can be established, as no DLS measurements were carried out [40]. Sunoqrot and colleagues reported that methoxy polyethylene glycol-b-poly(ε-caprolactone) nanoparticles (NPs) showed interaction with mucin molecules at any mucin:NP ratio (in *w*/*w*), from 0.25 (*w*/*w*) to 4.0 (*w*/*w*) via Schiff base and Michael addition reactions instead of electrostatic interactions, as the ζ potential of the mucin and NPs in the working conditions was close to neutrality. In such conditions, only bigger aggregates were formed at a mucin:NP ratio higher than 1.0 (*w*/*w*) [39]. Boya and colleagues also reported that the hydrodynamic size of metal NP coated with pluronic 127 increased 3 to 4 times after the interaction with mucin. However, no comparisons can be established with the results in the current work, as it was not possible to determine the mucin:metal NP ratio (*w:w*) used in the cited work [48].

The resulting interaction among mucin and o/w NCs found in this research work resembles the behaviour of other binary systems in which the ratio among the interacting species determines the size of the final nanostructures. Thus, at low and high interacting species ratios, bigger structures are formed, whilst there is an interacting species ratio range that results in the formation of nanometric structures [49]. Similarly, in this work, the o/w NCs can keep a nanometric size or aggregate to form higher structures depending on the mucin:o/w NC ratio. The fact that the resulting NCOM, which has mucin on their surface, can interact further with mucin molecules, can be related to the mucoadhesive properties of the formulation developed. Further studies that are currently ongoing in our research group will allow us to further elucidate the effectiveness and suitability of the developed o/w NCs as potential QS inhibition nanosystems.

## 5. Conclusions

1,3-dim-tolylurea (DMTU)-loaded CS-coated o/w NCs are proposed as a new oral antibacterial treatment because DMTU acts as a potent QS inhibitor that can be used to fight against bacteria biofilm formation, and CS has mucoadhesion and antibacterial properties. This study evaluated the stability of the proposed o/w NCs after the interaction with mucin molecules in suspension as unravelling the interactions between o/w NCs and mucins is essential for the rational design of drug delivery systems. The results show that o/w NCs are sensitive to the presence of mucin in the suspension. There is a range of mucin content in the media that results in the aggregation of the o/w NCs in a dynamic process. On the contrary, lower or higher mucin content in the media did not result in the aggregation of the o/w NCs, due to the appearance of repulsive electrostatic forces in their surfaces. The incorporation of DMTU to the o/w NCs resulted in a change in their conformation that resulted in the increase in their stability in the presence of mucin. Future work is required to evaluate the stability of the potential treatment developed in different conditions, such as simulated saliva, to determine the stability of the o/w NCs in a more realistic situation. It will be also important to study drug release from o/w NCs after interacting with mucin to evaluate the efficacy of the proposed drug delivery system.

## Figures and Tables

**Table 1:** Physico-chemical parameters of the mucin particles analysed by AF4.

	Region 1	Region 2
Mn (g/mol)	(2.24 ± 0.17) × 10^5^	-
Mw (g/mol)	(1.21 ± 0.02) × 10^6^	-
PD (Mw/Mn)	5.44	-
Rg (nm)	50.9 ± 1.3	252.0 ± 25.8
Mass eluted (%)	71.15	1.95

## Supplementary Materials

The following are available online at https://www.mdpi.com/2313-7673/5/3/36/s1, Table S1: DLS data analyses for mucin type III from Sigma-Aldrich; Table S2: ζ-potential and Z-average of NCOMs prepared at different mucin:B o/w NC ratios (in *w*/*w*) at time of synthesis (0 h) and 24 h after the synthesis event; Table S3: ζ-potential and Z-average of NCOMs prepared at different mucin:P o/w NC ratios (in *w*/*w*) at time of synthesis (0 h) and 24 h after the synthesis event; Figure S1. ζ-potential of NCOMs prepared at different mucin:o/w NC ratios (in *w*/*w*). B o/w NCs are plotted in grey and P o/w NCs are plotted in black. The ζ-potential values of the B o/w NCs and P o/w NCs are included at ratio 0,0.
