# Supplementary material for: Characterisation of the Interaction among Oil-In-Water Nanocapsules and Mucin
Source: Biomimetics (Basel). 2020 Jul 28;5(3):36. doi: 10.3390/biomimetics5030036 (PMC7559021; doi:10.3390/biomimetics5030036)
Supplement: Supplementary file 1 [file biomimetics-05-00036-s001.pdf]

Table S1. DLS data analyses for mucin type III from Sigma-Aldrich.

Concentration (mg/mL)	Hydrodynamic Radii						Intercept	ζ-potential
	Peak 1	%	Peak 2	%	Peak 3	%		
5.000	270.2±60.2	64.7	40.3±14.2	27.4	10.1±7.8	8.0	0.96	-16 ± 1
2.500	223.9±12.6	62.6	33.9±4.4	28.4	9.6±7.1	7.7	0.96	-19 ± 1
1.250	154.7±28.3	64.2	24.9±4.4	32.8	4.1±4.8	3.1	0.96	-24 ± 2
0.625	168.5±28.3	61.1	29.8±7.9	34.1	5.6±6.6	4.8	0.95	-22 ± 2
0.313	132.4±32.3	61.1	26.4±5.3	35.7	4.1±4.8	3.3	0.95	-25 ± 2
0.156	239.1±61.8	49.1	46.0±16.0	37.8	13.9±3.1	13.2	0.92	-27 ± 1
0.078	422.8±77.2	57.7	59.7±20.0	29.7	19.5±1.7	12.6	0.72	-29 ± 1

Table S2. ζ-potential and Z-average of NCOM prepared at different mucin:B o/w NC ratios (in w/w) at time of synthesis (0 h) and 24 h after the synthesis event.

Mucin:B o/w NC Ratio (in w/w)	Hydrodynamic Radii				Intercept	ζ-potential
**0 h**						
	Peak 1	%	Peak 2	%		
0	202±2	100	-	-	0.97	+40±1
0.08	408±123	100	-	-	0.82	+1 ± 0
0.17	500±39	97.7	41±70	2.3	0.85	-7 ± 0
0.34	923±107	71.5	172±15	27.9	0.94	-27 ± 0
0.67	1243±74	85.6	180±15	14.4	0.94	-29 ± 0
**24 h**						
Mucin:B o/w NC Ratio (in w/w)	Peak 1	%	Peak 2	%	Intercept	ζ-potential
0	202±2	100	-	-	0.97	+40±1
0.08	0 ± 0	0	-	-	0.80	-7 ± 0
0.17	1034.3±30.5	96.4	3459.0±2998.9	3.0	0.92	-19 ± 1
0.34	423.6±30.9	97.8	4931.3±182.0	2.2	0.94	-28 ± 1
0.67	308.8±28.9	96.9	35.2±30.7	3.1	0.94	-26 ± 1

Table S3. ζ-potential and Z-average of NCOM prepared at different mucin:P o/wNC ratios (in w/w) at time of synthesis (0 h) and 24 h after the synthesis event.

Mucin:P o/w NC ratio (in w/w)	Hydrodynamic Radii				Intercept	ζ-potential
**0 h**						
	Peak 1	%	Peak 2	%		
0.00	372±11	100	-	-	0.95	+54 ± 1
0.08	211±11	100	-	-	0.96	+39 ± 2
0.17	243±19	97.9	3114±2708	2.1	0.95	+13±0

0.34	415±36	93.2	1617±2671	5.6	0.95	-12±0
0.67	791±296	67.3	1777±2710	30.2	0.95	-24±1
1.34	1617±997	61.8	1762±2483	32.1	0.92	-26±1
2.68	473±21	90.8	4900±318	9.2	0.95	-27±1
**24 h**						
**Mucin:P o/w NC Ratio (in w/w)**	**Hydrodynamic Radii**				**Intercept**	**ζ-potential**
	**Peak 1**	**%**	**Peak 2**	**%**		
0.00	372±11	100	-	-	0.95	+54 ± 1
0.08	160±2	100	-	-	0.96	+22 ± 1
0.17	0±0	100	-	-	0.81	+2±0
0.34	510±29	99.4	1713±2966	0.57	0.93	-8±0
0.67	306±7	95.5	1676±2795	4.0	0.95	-23±1
1.34	367±42	98.0	4765±267	2.0	0.95	NM
2.68	522±135	79.2	1663±2642	17.8	0.94	NM

NM: not measured.

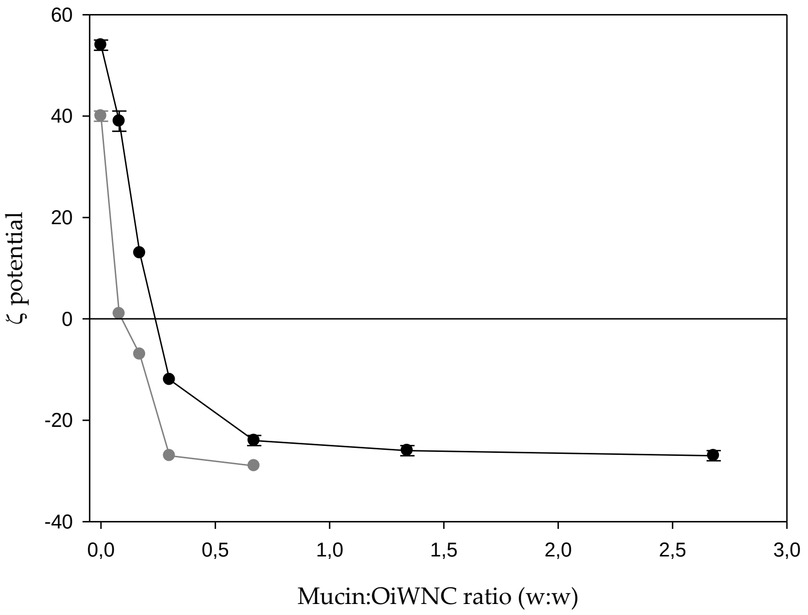

Figure S1. ζ-potential of NCOM prepared at different mucin:o/w NC ratios (in w/w). B o/w NC are plotted in gray and P o/w NC are plotted in black. The ζ-potential values of the B o/w NC and P o/w NC are included at ratio 0,0.
